# Predicting the future impact of climate change on the distribution of species in Egypt’s mediterranean ecosystems

**DOI:** 10.1186/s12870-025-06630-7

**Published:** 2025-05-15

**Authors:** Ahmed R. Mahmoud, Emad A. Farahat, Loutfy M. Hassan, Marwa Waseem A. Halmy

**Affiliations:** 1 Botany and Microbiology Department, Faculty of Science, Helwan University, P.O. Box: 11795, Helwan, Egypt; 2 Department of Environmental Sciences, Faculty of Science, Alexandria University, P.O. Box: 21511, Alexandria, Egypt

**Keywords:** Habitat loss, Ensemble model, MaxEnt, Biodiversity, Distribution models, Coastal deserts

## Abstract

As climate change accelerates, it may significantly alter species distributions and endanger many species. The use of species distribution modeling (SDM) has become increasingly vital for assessing the likely effects of climatic changes on biodiversity. This approach is especially relevant as our understanding of environmental shifts and their ecological implications deepens. SDMs are frequently employed to forecast future shifts in species’ geographic ranges, estimate extinction risks, evaluate the effectiveness of existing conservation areas, and prioritize conservation efforts. The urgency of these assessments is highlighted by the fact that the Mediterranean area is heating up 20% quicker than the universal average. Given that species have varying ecological tolerances and attributes, their biological responses to environmental changes are likely to differ significantly. This study aimed to assess the potential future distribution of three native Mediterranean species— *Thymelaea hirsuta* (L.) Endl., *Ononis vaginalis* Vahl, and *Limoniastrum monopetalum* (L.) Boiss.—under two GCMs of HadGEM3-GC31-LL and IPSL-CM6A-LR for the periods of 2060s and 2080s and two Shared Socioeconomic Pathway (SSP 1-2.6 and SSP5-8.5), comparing the use of MaxEnt and ensemble modelling techniques in predicting the impact of future climatic changes on these species’ distribution. The results indicated that there are high similarities and agreement between MaxEnt and the ensemble models’ outputs. The two modelling techniques exhibited excellent fits and performance. The distribution range of *T. hirsuta* and *O. vaginalis* will expand and migrate to the northwest direction of the Mediterranean coast of Egypt, while *L. monopetalum* will contract. The insights gained from species distribution modeling could guide future conservation efforts and promote the sustainable use of the studied species in the arid coastal environments of the Mediterranean region.

**Clinical trial number** Not applicable.

**Supplementary Information:**

The online version contains supplementary material available at 10.1186/s12870-025-06630-7.

## Introduction

Extreme weather conditions, including drought and heat waves that are probably brought on by climate change, frequently have a profound impact on plant species and ecosystems. Variations in species’ ecological tolerance and attributes are likely the underlying factors causing different biological responses to these environmental changes [1].

Climatic changes have led to increased temperatures and more frequent droughts in various regions worldwide [2]. The climate change of the Mediterranean region and its sub-regions exhibits a continued increase in air and sea surface temperature, as well as projected changes in rainfall pattern [3]. If the combination of climate change and other human-induced changes (e.g., land use, pollution, and resources overexploitation) continues, the resilience of various ecosystems will be surpassed [2], altering their structure and function [4]. Climate change might affect the potential geographic spread of many plant species due to fluctuations in temperature and rainfall regimes [5].

Species distribution models (SDMs) have been widely employed for predicting shifts in species distribution due to climatic changes; and for creating ecological suitability mapping under existing and future conditions [6, 7]. SDMs are frequently applied for forecasting the change in the species geographic distribution of under different climatic conditions. Using different bioclimatic, topographic, edaphic, and habitat variables can dependably estimate the species ecological niche and predict species range changes under different climatic conditions [8].

MaxEnt is amongst the most widely used SDM approaches. The advantage of using MaxEnt is that it can be trained on presence-only data and works well with low-size samples [9, 10]. However, the employment of ensemble models is recommended over relying on a single modeling approach to evaluate the role of climatic changes in causing changes in species geographic extent [11, 12]. Additionally, ensemble modeling techniques, which combine results from multiple models, are recommended for assessing species range shifts under climate change scenarios [11, 12]. The use of ensemble modelling techniques was preferred over the use of the outcomes from a single modelling approach to evaluate the impact of climate changes on the range shift of species. The ensemble modelling techniques provide more robust and accurate results and avoid overfitting of the model [11]. Besides, they minimize the prediction generalization errors and reduce overfitting when modelling rare species. Ensemble modelling is considered a better alternative to single models for future climate projection modelling with large numbers of species [13]. Research has demonstrated that ensemble techniques offer significant advantages compared to using a single algorithm [11, 14, 15]. The utilization of the ensemble modelling is believed to lower the uncertainty and enhance robustness, while avoiding model overfitting [14, 16, 17].

Three perennial plant species namely *Thymelaea hirsuta* (L.) Endl. (Family Thymelaeaceae), *Limoniastrum monopetalum* (L.) Boiss (Family Plumbaginaceae) and *Ononis vaginalis* Vahl (Family Fabaceae) are native to the Mediterranean region. The Egyptian Mediterranean coast stretches for 970 km along the Mediterranean Sea. The western Mediterranean coastal region is characterized by several types of ecosystems, entirely covering the northern territory of Egypt from El-Sallum eastward to Rafah. It is considered among the lengthiest Mediterranean shorelines in North Africa [18, 19]. Climatically, it is the least arid belt in Egypt. The region has the highest species richness in Egypt [20] and is characterized by landforms that vary in edaphic and topographic characteristics such as oolitic dunes, saline depressions, salt marshes, rocky ridges, sand formations, and sand flat sheets or plains. Drought, salinity, soil erosion, and anthropogenic disturbances including urban development, agriculture, and quarrying pose major threats to the natural vegetation of the western Mediterranean coast [19, 21]. Halmy (2019) has recorded a decline in species richness and an increase in anthropogenic factors that affect species abundance as the main trend associated with the changes in land use/cover of the region [22].

Hence, the present study sought to evaluate the potential effect of climatic changes, based on two shared socio-economic pathways (SSPs), on the projected suitable habitat and potential geographic extent of the investigated species. This assessment was conducted under both full and restricted dispersal scenarios, utilizing multiple modeling techniques, including MaxEnt and ensemble models.

## Materials and methods

### Study area and studied species

**Fig. 1:**
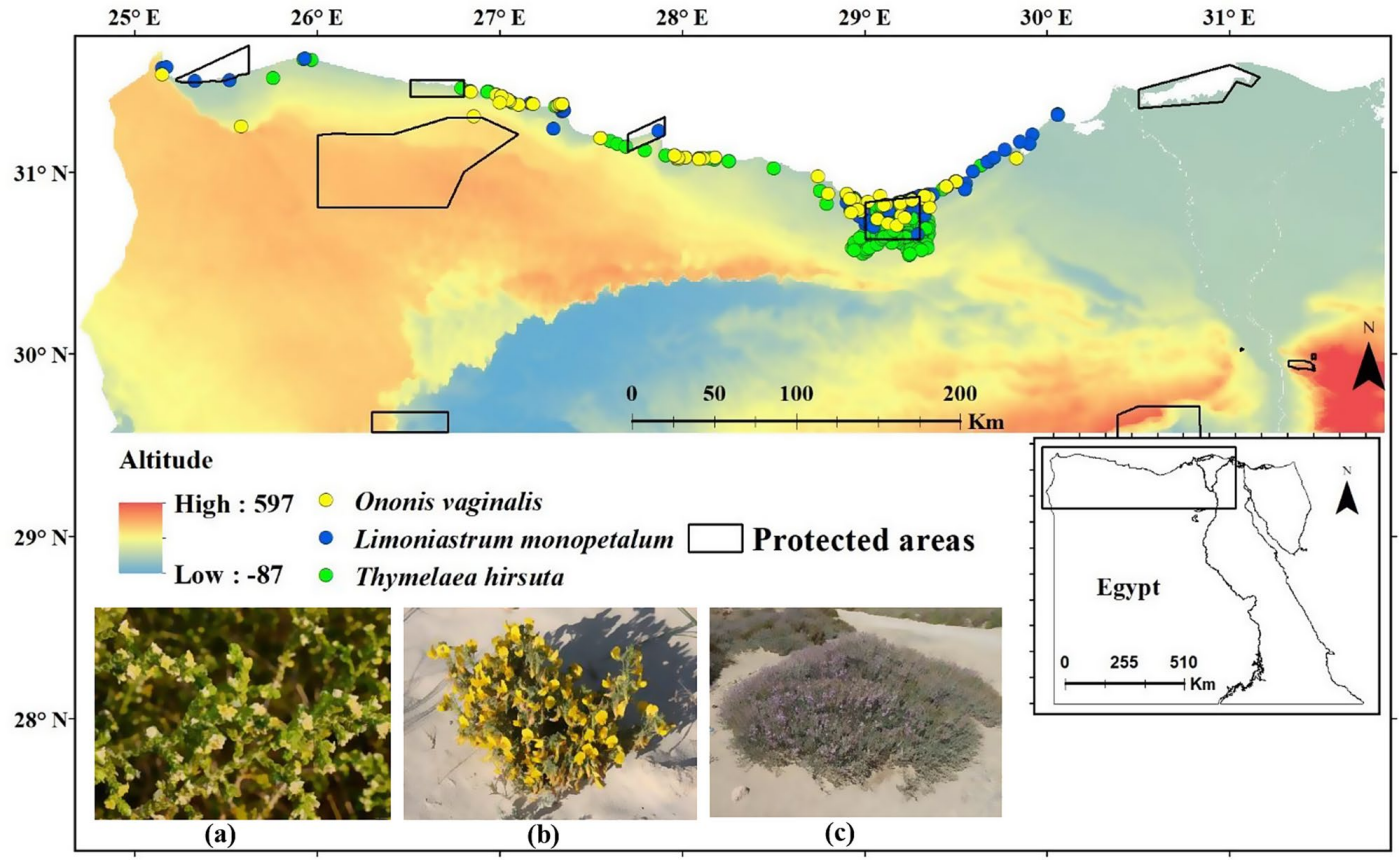
The surveyed study area illustrating the locations of the collected occurrence records of the investigated species (**a**) *Thymelaea hirsuta*, (**b**) *Ononis vaginalis* and (**c**) *Limoniastrum monopetalum*, the study area covers 175,018 km^2^ of total area of Egypt

This study was conducted along the northern coastline of Egypt’s western Mediterranean region (Fig. 1), which is situated in the country’s least arid zone, between latitudes 29.57 and 31.67 N and longitudes 24.71 and 31.85 E. The study area covers 175,018 km^2^ of total area of Egypt. This area is recognized as the region with the highest species richness in Egypt [20] and is distinguished by diverse landforms with varying edaphic and topographic characteristics. Climatically, the western Mediterranean coastal land is part of the dry arid climatic zone of Koppen’s (1931) [23], with a mean annual precipitation that ranges between 100 and150 mm/year [24]. The rainy season is short, and occurs mostly during winter from November to April, but may extend to May. Little precipitation occurs during the rest of the year [25]. The annual mean maximum temperature ranges from 23.8 °C to 25.3 °C, while the annual mean minimum temperature ranges from 13.3 °C to 15.1 °C [26]. The study area encompasses the distribution regions of three Mediterranean endemic species that serve as key indicators of the major habitats in Egypt’s coastal deserts. *Thymelaea hirsuta* (L.) Endl. (Family: Thymelaeaceae), *Ononis vaginalis* Vahl (Family: Fabaceae), and *Limoniastrum monopetalum* (L.) Boiss (Family: Plumbaginaceae). The three species are threatened by the reduction in their populations, in addition to the exponential loss and degradation of their natural habitats brought on by recent human activities such as urban development, pollution, and deforestation [21, 22]. The field work follows a latitudinal transect of the Mediterranean coast western section that extends for nearly 550 km from Abu Qir to El-Sallum (29.57°-31.67° N and 24.71°-31.85° E) crossing different types of ecosystems. Thus, the field survey covers a wide range of habitats and vegetation types including sand dunes (coastal calcareous and inland siliceous); rocky ridges, inland plateau, non-saline depressions, and saline depressions; and wadis [26]. Besides these habitats, two more habitats were described by Batanouny (1973), namely the uncultivated desert areas and the sand plains [27].

Data showing the location of the existing protected area in the north part of Egypt were obtained from the Protected planet database [28]. These data comprise the boundaries of protected areas. The obtained data comprise 11 protected areas meeting the Protected Area Management Categories I–VI of the IUCN [29], with a total area of approximately 8984 km2 (Fig. 1).

The conservation status of these species according to The IUCN Red List of Threatened Species has not been assessed; therefore, their global conservation status is currently unknown. Their conservation status in Egypt is also not known, though they have been reported as highly threatened due to habitat loss caused by human activities, including the establishment of tourist resorts and quarrying activities (Bidak et al., 2015).All procedures involving field research and plant collection were conducted in full compliance with the institutional, national, and international regulations.

Identification of the plant material used in the study was performed by Prof. Dr./ Loutfy Mohsen Hassan professor of plant ecology and flora at Faculty of Science- Helwan University – Botany and Microbiology department. Some of the collected samples were kept in Helwan University Herbarium (HEU) (vouchers no 0111064 to 0111079 five vouchers for each species).

### Species occurrence data collection

A total of 449 occurrence records for the three species were collected during field surveys conducted from July to September 2021. Each observation was georeferenced using a Global Positioning System (Garmin GPSMAP 64sx), capturing both latitude and longitude coordinates. The coordinates of these points were included in the geographic information system (GIS) domain and made ready for use in the subsequent analysis. The field surveys covered the main habitats in the western sector of the Mediterranean coastal region, where plots were selected randomly to represent the major physiographic variations. Most of the records, 310 occurrence points (69%), were for *T. hirsuta*, while 65 occurrence points (15%) were for *O. vaginalis*, and 74 occurrence points (16%) were for *L. monopetalum* (Fig. 1).

### Environmental data

A total of 35 environmental variables representing factors important for plant distribution were initially selected (Supplementary Material Table S1). The primary variables used in the distribution models of the studied species can be categorized into four main groups: (1) bioclimatic that were acquired from the World Climate Database [30] (http://worldclim.org/version2) version 2.1 at 30 arc-seconds (1 km) spatial resolution, (2) topographic that is represented by elevation data that was downloaded from the USGS Dataset [31] (https://www.usgs.gov), (3) edaphic that were represented by nine soil variables from the SoilGrids dataset [32] (https://soilgrids.org), and (4) Habitat-type data were developed according to the habitat classification scheme of Egypt prepared by Harhash et al. (2015) [33]. To account for maritime influences due to proximity to the sea the distance to the coast was also included as a factor. The proximity to sea was found to influence the distribution of the species in the region. The distance from the coast reflect the variation in favorable microclimatic conditions, moisture availability, and even human-mediated disturbances in the region (e.g., resorts, roads), which can influence the establishment and spread of species [21]. Finally, all these data layers representing the environmental variables were maintained at 30 arc-second (~ 1 km) resolutions and were cropped to the spatial extent of the investigated area (Fig. 1). The forecasting of the species distributional shifts investigated was conducted under the two GCMs of HadGEM3-GC31-LL and IPSL-CM6A-LR for the periods of 2060s (average of 2041 to 2060) and 2080s (average of 2061–2080) and two Shared Socio-economic Pathways (the SSP1-2.6 and SSP5-8.5). This was performed to depict the variations in the projections by the GCMs and to have insights into the potential chances of range shift for the investigated species under different emission scenarios both in the short-term and the long-term. The two global climate models were obtained from the most recent sixth level of the Coupled Model Intercomparison Project (CMIP6) (https://www.worldclim.org/data/cmip6/cmip6_clim30s.html).

Preparative exploratory data analyses, including a normality test, correlation analysis, and multicollinearity statistics, were carried out to inspect the relevance of the environmental attributes. To detect the multicollinearity and identify the influential variables to be used in modelling the distributional extent of the investigated species, variance inflation factors (VIFs) were calculated. Variables with a VIF more than five were neglected as their contributions were negligible [34]. VIF was implemented using “usdm” package [35]. The two algorithms vifcor and vifstep of the usdm R package were used within the framework of R 4.2.1[36, 37].

The geographical extent of the investigated species was modelled under current climate conditions with a reduced set of variables of the initial set of variables (Supplementary Material Table S2) after accounting for the collinearity by applying Variance Inflation Factor analysis.

### Ecological niche modelling and evaluation

Species occurrences and environmental variables were used to model the geographic distribution of the studied species using MaxEnt version 3.4.4 [9] and an ensemble modeling approach. MaxEnt is a machine learning method that estimates the suitability of an area through assessing the probability distribution of maximum entropy. An ensemble of three modelling algorithms were chosen for constructing the ensemble species distribution model. The modelling algorithms included the generalized linear model (GLM: [38]) as parametric technique, the Boosting Regression Trees (BRT: [39, 40]) and the random forests (RF: [41, 42]) as non-parametric machine-learning techniques. The selected model approaches are characterized by high stability and transferability compared to other models [13, 43–46]. Furthermore, GLM and RF behave best on both cross-validation and external validation [13]. About 70% of the occurrence records for each species were selected at random to calibrate the models, while the remaining 30% were used for accuracy assessment and performance evaluation [47]. Model performance was assessed using the area under the receiver operating characteristic curve metric (AUC) [48]. Several accuracy metrics were also estimated to assess model performance, including overall accuracy, sensitivity, specificity, and true skill statistics (TSS). Additionally, the Area Under the Curve (AUC) of the Receiver Operating Characteristic was a key metric for performance evaluation.

### Geospatial analysis

The prediction of the established models represents the species probability of occurrence, with values from 0 to 1, where 0 indicates the unsuitable area for the species and 1 indicates the optimal area for the occurrence of the modeled species. To obtain maps of existence areas for the studied species, the continuous probability maps produced as outputs of the established models were converted to binary maps representing the potential presence and absence areas for the investigated species.

Additionally, to classify the study area with regard to its suitability for the investigated species, the probability maps produced as outputs of the established models were classified based on the values of the probability of occurrence into four levels of habitat suitability, high, medium, low, and unsuitable (Table 1). The reclassification function in the Spatial Analyst Tools withing the framework of ArcGIS 10.2 (Environmental Systems Research Institute 2013) was used for conducting the classification.

**Table 1:** Habitat suitability classes and the probability of occurrence range for the investigated species

Species	Suitability Class			
	Unsuitable	Low	Moderate	High
*Thymelaea hirsuta*	< 0.16	0.161-0.30	0.301-0.60	> 0.601
*Ononis vaginalis*	< 0.10	0.101–0.35	0.351–0.65	> 0.651
*Limoniastrum monopetalum*	< 0.10	0.101–0.35	0.351–0.65	> 0.651

To assess changes in the geographic extent of suitable habitat as loss, gain, or persistence under future scenarios, the “Map Algebra” functions within ArcGIS 10.2 were applied to the binary maps generated from the transformed present and future habitat suitability probability maps. The estimation of the shifts in suitable habitat distribution between present and future scenarios for the investigated time intervals were calculated using SDM Toolbox v.2.5 within the ArcGIS 10.2 framework. This process produced potential change maps for each time interval under the different scenarios. By analyzing the changes in the potential habitat distributional extent using binary maps for the studied species under various climate scenarios, the tendency for geographical shifts in each species distribution was examined.

## Results

### Model performance and evaluation

The geographical extent of the investigated species were modelled with a reduced set of variables of the initial set of variables (Table S2 and S3) after accounting for the collinearity by applying Variance Inflation Factor analysis. The study assessed the predictive power of the MaxEnt and ensemble models in modelling the distributional extent of the investigated species. The outcomes of the two modeling techniques demonstrated high predictive accuracy, with a mean AUC value exceeding 0.98 and mean TSS value lager than 0.85 for all models of all species (Table 2).

**Table 2:** Accuracy measures used for the evaluation of the models of the potential distributional extent of the investigated species

Species	Model	Overall Accuracy (%)	Sensitivity (%)	Specificity (%)	TSS	AUC
*T. hirsuta*	MaxEnt	97 ± 0. 000	99 ± 0.006	97 ± 0.000	0.96 ± 0.006	0.98 ± 0.001
	Ensemble average	95 ± 0.016	99 ± 0.060	95 ± 0.050	0.93 ± 0.014	0.99 ± 0.004
*O. vaginalis*	MaxEnt	97 ± 0. 00	88 ± 0.13	97 ± 0.00	0.85 ± 0.12	0.98 ± 0.01
	Ensemble average	95 ± 0.01	96 ± 0.01	94 ± 0.01	0. 90 ± 0.05	0.98 ± 0.01
*L. monopetalum*	MaxEnt	96 ± 0.003	98 ± 0.034	96 ± 0.003	0.94 ± 0.036	0.98 ± 0.001
	Ensemble average	98 ± 0.010	98 ± 0.030	96 ± 0.010	0.94 ± 0.024	0.98 ± 0.010

### Projected distribution of suitable habitats under future climate scenarios

**Fig. 2:**
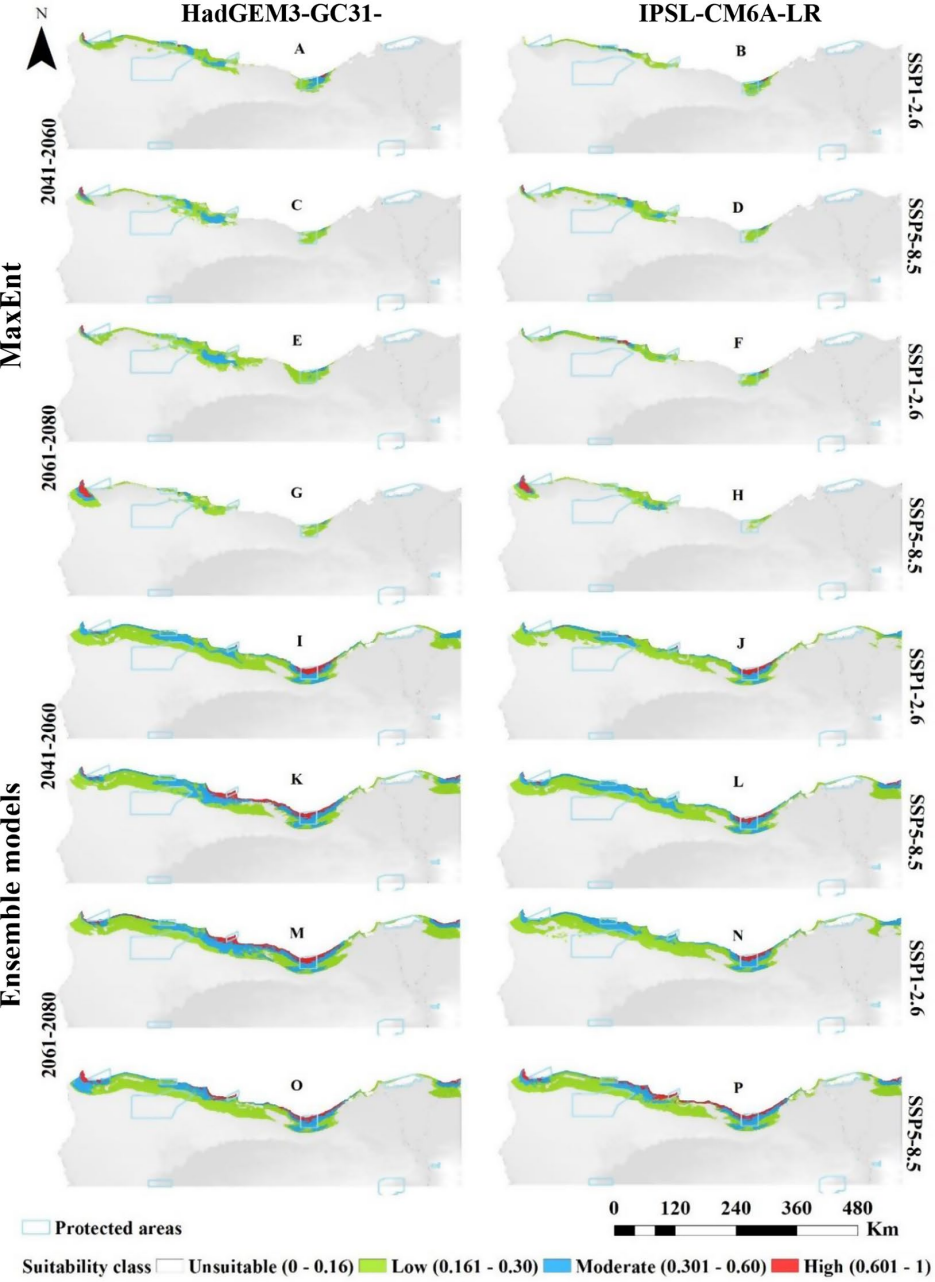
Predicted distribution of *Thymelaea hirsuta* by MaxEnt and Ensemble model based on two warming scenarios, SSP1-2.6 (**A**, **B**, **E**, **F**, **I**, **J**, **M** & **N**) and SSP5-8.5 (**C**, **D**, **G**, **H**, **K**, **L**, **O** & **P**) of the HadGEM3-GC31-LL and the IPSL-CM6A-LR general climate model by the period 2041–2060 (**A**, **B**, **C**, **D**, **I**, **J**, **K** & **L**) and 2061–2080 (**E**, **F**, **G**, **H**, **M**, **N**, **O** & **P**)

The projected distribution of *T. hirsuta*, under two Shared Socio-economic Pathways (SSP1-2.6 and SSP5-8.5) of the HadGEM3-GC31-LL general climate model for the near and far future (2060s and 2080) was compared to the current distribution. MaxEnt modeling estimated that the suitable habitat would increase in near and far future, particularly under SSP1-2.6, reaching 7,940 km² (4.54% of study area) by the 2060s and 9,894 km² (5.65% of the total study area) by 2080. Conversely, predictions under the IPSL-CM6A-LR GCM climate scenario indicated a reduction in suitable habitat by 2060s, predicting a shrink of the area to 3,758 km² (2.15% of the study area) under SSP1-2.6 and 2,800 km² (1.31%) under SSP5-8.5. In the far future (2080s), however, the model projected a rise in suitable habitat to 5,048 km² (2.88%) for SSP1-2.6 and 5,100 km² (2.91%) for SSP5-8.5 (Fig. 2; Table 3).

Ensemble model projections for *T. hirsuta* showed an overall increase in suitable habitat across both scenarios and time periods. In the far future (2080s) the increase projected to be more pronounced, with suitable habitat expanding by 23,891 km² (13.65%) under SSP1-2.6 and by 21,733 km² (12.42%) under SSP5-8.5. The model of IPSL-CM6A-LR scenario predicted habitat increases across all SSPs for the far and near future, with significant increase of 21,744 km² (12.44%) in the 2060s and 22,110 km² (12.63%) in the 2080s (Fig. 2; Table 3).

**Fig. 3:**
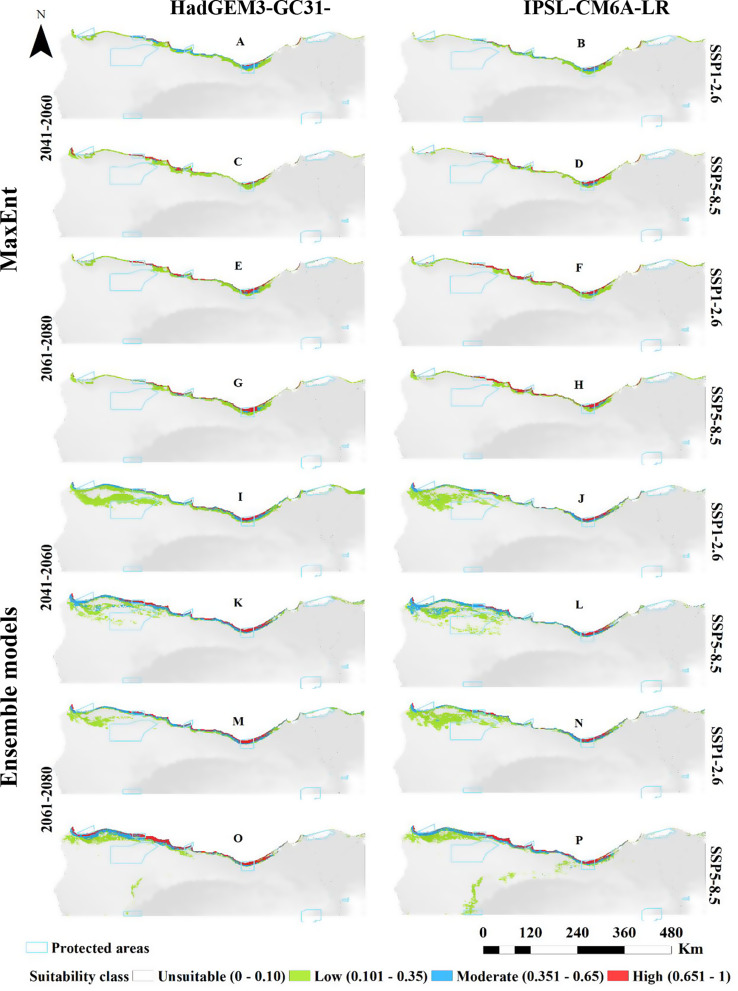
Predicted distribution of *Ononis vaginalis* by MaxEnt and Ensemble model based on two warming scenarios, SSP1-2.6 (**A**, **B**, **E**, **F**, **I**, **J**, **M** & **N**) and SSP5-8.5 (**C**, **D**, **G**, **H**, **K**, **L**, **O** & **P**) of the HadGEM3-GC31-LL and the IPSL-CM6A-LR general climate model by the period 2041–2060 (**A**, **B**, **C**, **D**, **I**, **J**, **K** & **L**) and 2061–2080 (**E**, **F**, **G**, **H**, **M**, **N**, **O** & **P**)

For *O. vaginalis*, projections under the HadGEM3-GC31-LL climate scenario (CCSM6) for the near (2060s) and far (2080s) future showed a progressive increase in the suitable habitat compared to current distribution (Fig. 3; Table 3). At both emission scenarios (SSP1-2.6 and SSP5-8.5), habitat suitability increased with climate warming. By the 2060s, suitable habitat would increase by 5,806 km² under SSP1-2.6 and by 5,640 km² under SSP5-8.5. A similar pattern was observed for the 2080s, with a rise of 5,389 km² at SSP1-2.6 and 5,889 km² at SSP5-8.5. Predictions under the SSP1-2.6 scenario of the IPSL-CM6A-LR GCM model for the 2060s projected a reduction in suitable habitat to 4,820 km², but by 2080, it was expected to increase to 5,878 km². Under SSP5-8.5, suitable habitat would decrease to 4,662 km² by the 2060s, followed by an expansion to 5,444 km² by 2080. Long-term projections indicated a reduction in low and moderate suitability areas, but high suitability areas would expand relative to the current distribution.

Ensemble models for *O. vaginalis* under the HadGEM3-GC31-LL climate scenario also projected increases in suitable habitat under the two SSP scenarios. In the far future (2080s), suitable habitat would reach 8,699 km² under SSP1-2.6 and 10,677 km² under SSP5-8.5. The IPSL-CM6A-LR scenario projected a continuous increase in suitable habitat, reaching 10,644 km² by the 2060s under SSP1-2.6, with further increases across all suitability categories by 2080 (11,021 km²). Under SSP5-8.5, the suitable area was projected to increase to 12,479 km² by 2080, showing expansions across high, moderate, and low suitability areas (Fig. 3; Table 3).

For *L. monopetalum*, MaxEnt projections under the HadGEM3-GC31-LL climate scenario for SSP1-2.6 showed a decline in the suitable habitat compared to current predictions, with reductions of 2,680 km² by the 2060s and 2,732 km² in the far future (2080s). Under SSP5-8.5, suitable habitat would decrease to 2,555 km² by the 2060s and 840 km² in the far future (2080s). Predictions under the IPSL-CM6A-LR GCM for SSP1-2.6 revealed a projected reduction in suitable habitat to 3,169 km² by the 2060s, with a slight increase to 4,171 km² by 2080. However, the majority of the remaining areas would have low suitability.

**Fig. 4:**
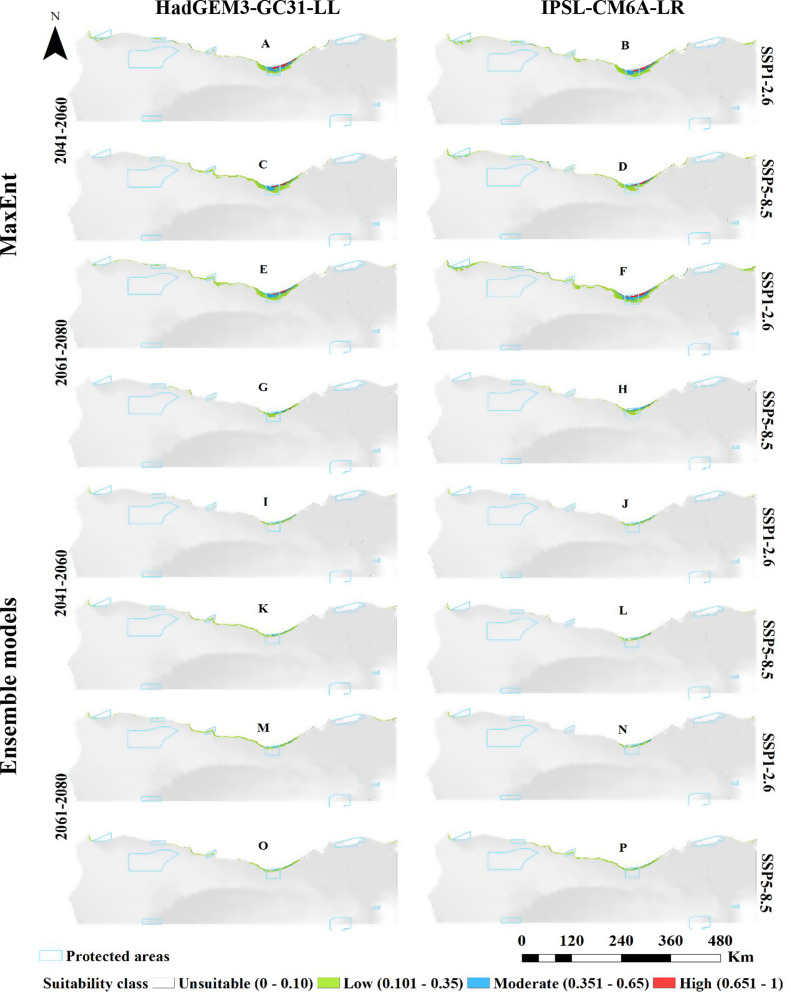
Predicted distribution of *Limoniastrum monopetalum* by MaxEnt and Ensemble model based on two warming scenarios, SSP1-2.6 (**A**, **B**, **E**, **F**, **I**, **J**, **M** & **N**) and SSP5-8.5 (**C**, **D**, **G**, **H**, **K**, **L**, **O** & **P**) of the HadGEM3-GC31-LL and the IPSL-CM6A-LR general climate model by the period 2041–2060 (**A**, **B**, **C**, **D**, **I**, **J**, **K** & **L**) and 2061–2080 (**E**, **F**, **G**, **H**, **M**, **N**, **O** & **P**)

Ensemble modeling for *L. monopetalum* under the HadGEM3-GC31-LL scenario showed a continued decrease in suitable habitat across both SSP1-2.6 and SSP5-8.5, with total suitable areas shrinking to 652 km² by the 2060s and 1,779 km² by the 2080s under SSP1-2.6, and to 1,231 km² by the 2060s and 977 km² by in the far future (2080s) under SSP5-8.5. Under the IPSL-CM6A-LR GCM model, the suitable extent for *L. monopetalum* would decrease to 628 km² by the 2060s and 571 km² by 2080 under SSP1-2.6, while under SSP5-8.5, the area would increase to 1,333 km² by 2080 (Fig. 4; Table 3).

**Table 3:** The area (in km^2^) of the suitable habitat for the explored species currently and under future climate scenarios using MaxEnt and ensemble models predictions

Species	Model	Suitability class	Current	HadGEM3-GC31-LL				IPSL-CM6A-LR			
				2041–2060		2061–2080		2041–2060		2061–2080	
				SSP1-2.6	SSP5-8.5	SSP1-2.6	SSP5-8.5	SSP1-2.6	SSP5-8.5	SSP1-2.6	SSP5-8.5
*T. hirsuta*	MaxEnt	Unsuitable	170,189	167,078	168,642	165,124	169,149	171,260	172,718	169,970	169,918
		Low	1898	5904	4797	7960	4520	3040	1901	3961	3864
		Moderate	2379	1820	1460	1848	895	621	287	825	829
		High	552	216	119	86	454	97	112	262	407
	Ensemble	Unsuitable	169,476	153,604	153,254	151,127	153,285	158,304	153,274	154,410	152,908
		Low	3217	14,388	12,581	13,754	13,739	11,417	14,011	13,935	13,986
		Moderate	1192	6159	7174	7781	6282	4560	6913	6094	5795
		High	1133	867	2009	2356	1712	737	820	579	2329
*O. vaginalis*	MaxEnt	Unsuitable	169,484	169,212	169,378	169,620	169,129	170,198	170,356	169,140	169,574
		Low	3843	3773	4225	3466	3940	3385	3265	3872	3257
		Moderate	1279	1600	415	718	741	1121	493	578	577
		High	412	433	1000	1214	1208	314	904	1428	1610
	Ensemble	Unsuitable	170,641	161,607	164,387	166,328	164,341	164,374	163,412	163,997	162,539
		Low	2929	10,231	5253	4438	4802	7627	6393	7891	7123
		Moderate	1139	2318	3744	2790	3314	2156	4288	2248	3459
		High	309	862	1634	1462	2561	861	925	882	1897
*L. monopetalum*	MaxEnt	Unsuitable	168,489	172,338	172,463	172,286	174,178	171,849	172,718	170,847	173,943
		Low	4626	1798	2025	2150	682	2386	1901	3134	857
		Moderate	1455	627	387	439	142	578	287	786	203
		High	448	255	143	143	16	205	112	251	15
	Ensemble	Unsuitable	170,068	174,366	173,787	173,529	174,041	174,390	174,426	174,447	173,685
		Low	3532	537	1127	1652	884	516	511	471	1232
		Moderate	1153	115	104	127	93	112	81	100	101
		High	265	0	0	0	0	0	0	0	0

### Projected effects of Climatic changes

**Fig. 5:**
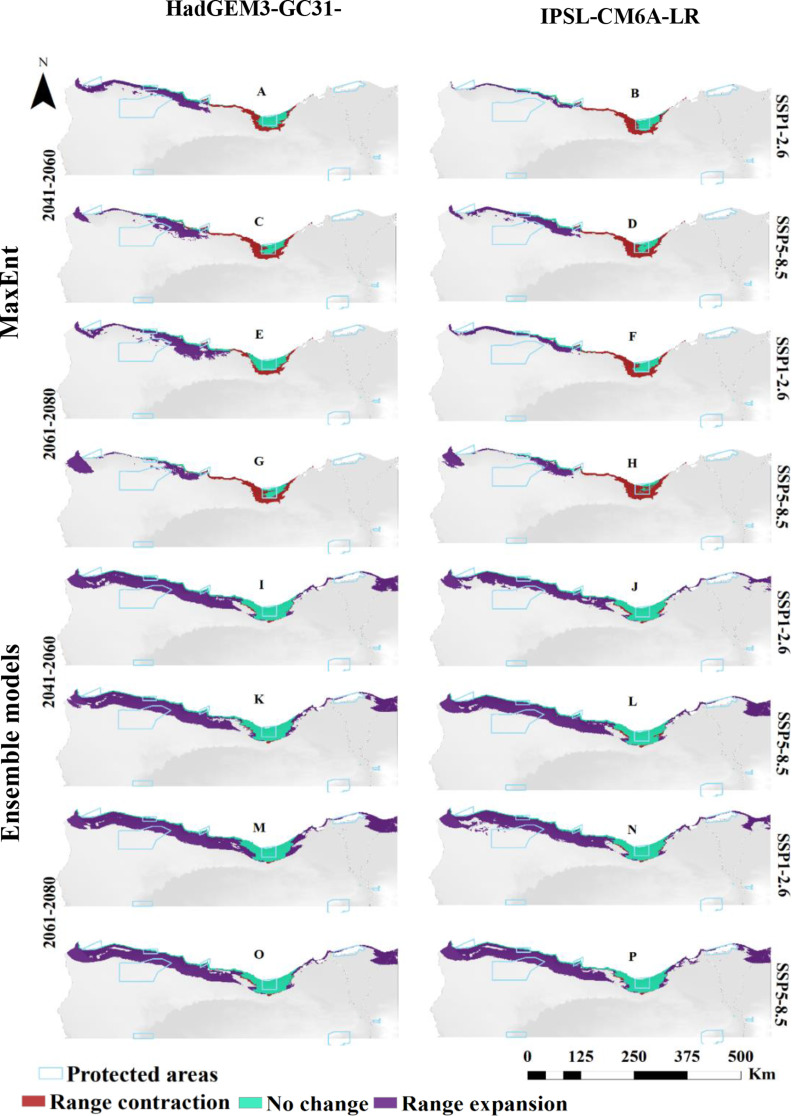
Predicted range expansion/contraction of *Thymelaea hirsuta* under the investigated warming scenarios resulting from the MaxEnt and Ensemble models. The change in distribution between current and future climate of the HadGEM3-GC31-LL and the IPSL-CM6A-LR general climate model under SSP1-2.6 (**A**, **B**, **E**, **F**, **I**, **J**, **M** and **N**) and SSP5-8.5 (**C**, **D**, **G**, **H**, **K**, **L**, **O** and **P**) scenarios by the period 2041–2060 and 2061–2080

MaxEnt model outcomes revealed that the geographic extent of *T. hirsuta* is projected to shift under different climate models and Shared Socio-economic Pathways (SSPs) in both the near and far future. Under the HadGEM3-GC31-LL model, for the periods 2041–2060 and 2061–2080, expansion is expected west of the investigated area, with contractions in the middle and eastern regions. Specifically, by 2060, the range is expected to shrink by 2,178 km² under SSP1-2.6 and 3,166 km² under SSP5-8.5, while expansions of 5,289 km² and 4,714 km² are projected in the northwest. By 2080, further contraction is projected, but with expansions of 6,660 km² and 4,562 km² under the two SSPs (Fig. 5, and 8).

Similarly, the IPSL-CM6A-LR model also predicts both contractions and expansions for *T. hirsuta*. By 2060, contraction is expected across 2,775 km² to 3,201 km², while expansions between 1,704 km² and 4,448 km² are projected. By 2080, contraction will continue, but expansions by about 4,003 km² are also predicted.

The ensemble models confirm these general trends, with more expansion predicted in the east and south in the investigated area. Contraction in the central region and expansion in the northwestern region is consistent across both models. By 2080, under HadGEM3-GC31-LL, expansion could reach 18,428 km² under SSP1-2.6, with less contraction than in earlier periods.

**Fig. 6:**
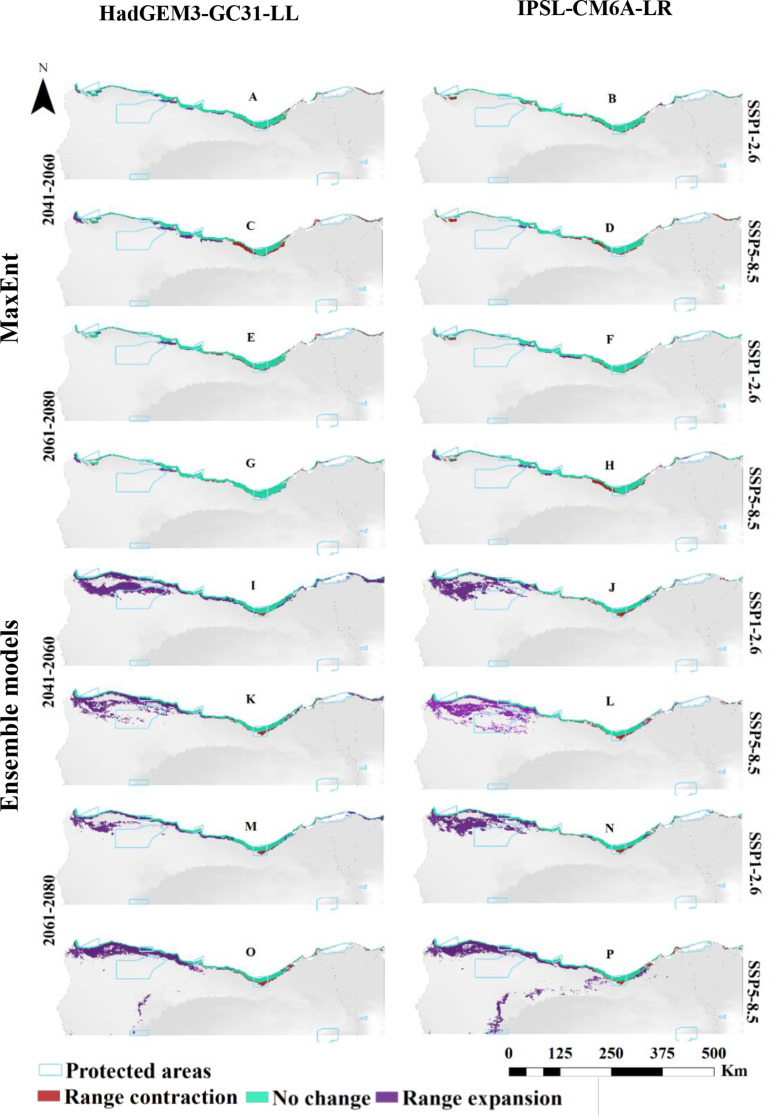
Predicted range expansion/contraction of *Ononis vaginalis* under the investigated warming scenarios resulting from the MaxEnt and Ensemble models. The change in distribution between current and future climate of the HadGEM3-GC31-LL and the IPSL-CM6A-LR general climate model under SSP1-2.6 (**A**, **B**, **E**, **F**, **I**, **J**, **M** and **N**) and SSP5-8.5 (**C**, **D**, **G**, **H**, **K**, **L**, **O** and **P**) scenarios by the period 2041–2060 and 2061–2080

For *O. vaginalis*, the distribution pattern is also projected to change, showing contraction in the suitable extent compared to current conditions. Under HadGEM3-GC31-LL, by 2060, the species is anticipated to lose 555 km² (SSP1-2.6) and 1,205 km² (SSP5-8.5) of its current habitat, but expansion will occur in the northwesterly by 828 km² and 1,311 km², respectively. This trend highlights a range shift, balancing both contraction and expansion under the investigated climate scenarios (Fig. 6, and 8).

**Fig. 7:**
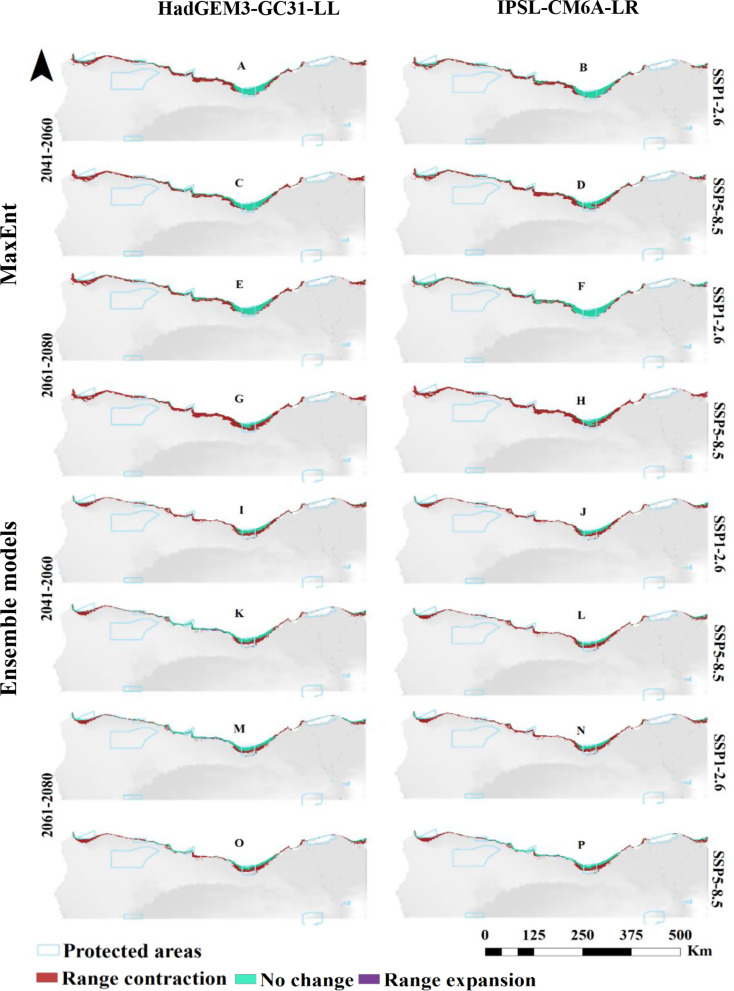
Predicted range expansion/contraction of *Limoniastrum monopetalum* under the investigated warming scenarios resulting from the MaxEnt and Ensemble models. The change in distribution between current and future climate of the HadGEM3-GC31-LL and the IPSL-CM6A-LR general climate model under SSP1-2.6 (**A**, **B**, **E**, **F**, **I**, **J**, **M** and **N**) and SSP5-8.5 (**C**, **D**, **G**, **H**, **K**, **L**, **O** and **P**) scenarios by the period 2041–2060 and 2061–2080

By 2080, a contraction in the species range of *O. vaginalis* is projected, with 639 km² and 307 km² under SSP1-2.6 and SSP5-8.5 scenarios, respectively. Expansion is expected in the northwest, estimated at 504 km² under SSP1-2.6 and 662 km² under SSP5-8.5, with larger expansion under SSP5-8.5. Overall, more contraction is anticipated under SSP5-8.5 by 2060, while expansion is more substantial by 2060 than 2080 under both scenarios.

The IPSL-CM6A-LR model projects a contraction of 906, 1,148, 391, and 1,001 km² under SSP1-2.6 and SSP5-8.5 for 2041–2060 and 2061–2080, respectively. Expansion is expected at 193, 276, 735, and 911 km², mostly toward the northwest. SSP5-8.5 shows greater contraction and expansion compared to SSP1-2.6, with expansion increasing over time.

**Fig. 8:**
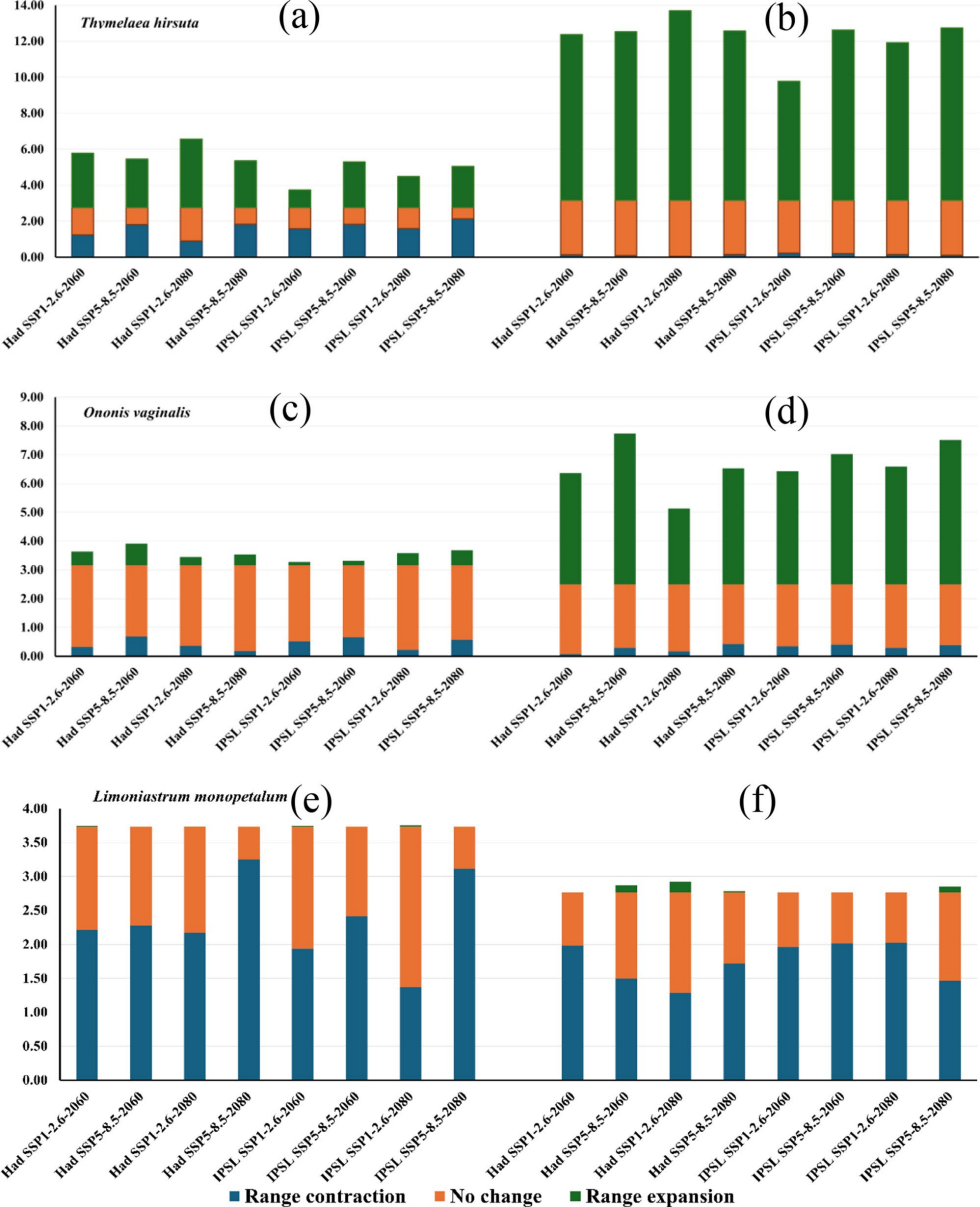
Percentage of the studied species range persistence, expansion, and contraction as predicted by MaxEnt ((**a**), (**c**) and (**e**)) and ensemble model ((**b**), (**d**) and (**f**)) under the scenarios SSP1-2.6 and SSP5-8.5 based of the HadGEM3-GC31-LL and IPSLCM6A-LR general climate model during the period 2041–2060 and 2060–2080

For *L. monopetalum*, a general contraction is predicted, particularly near the Mediterranean shoreline, in its suitable habitat. Under the HadGEM3-GC31-LL model, contraction by 3,877 and 3,983 km² is predicted for 2060 under SSP1-2.6 and SSP5-8.5, with slight to no expansion. By 2080, contraction is estimated at 3,799 and 5,689 km², with little to no expansion expected (Fig. 7, and 8).

The trends reveal that investigated scenarios will likely result in more habitat contraction, though some areas might experience expansion, especially under SSP5-8.5.

The probable range shift of the species under the two Shared Socio-economic Pathways (SSP1-2.6 and SSP5-8.5) of the IPSL-CM6A-LR general circulation model by 2060 and 2080 (Figs. 7 and 8) revealed that the current distribution area will contract by 3,388 km², 4,229 km², 2,395 km², and 5,454 km² under SSP1-2.6 (2041–2060), SSP5-8.5 (2041–2060), SSP1-2.6 (2061–2080), and SSP5-8.5 (2061–2080), respectively. In contrast, expansion will be minimal, with increases of 28 km², 0 km², 37 km², and 0 km² under these scenarios respectively.

Both the HadGEM3-GC31-LL and IPSL-CM6A-LR models suggest negligible to no expansion in suitable areas under various scenarios in both the short and long terms. Any potential expansion is probable to be northward. The contraction rate is anticipated to rise over time, reaching 83–87% of the present suitability area.

The ensemble model projections for *L. monopetalum* align with that of the MaxEnt model, showing a general trend of contraction in suitable areas relative to the current distribution pattern across different climate models and SSP scenarios.

For the HadGEM3-GC31-LL model, the current distribution area will contract by 3,463 km² and 2,621 km² under SSP1-2.6 and SSP5-8.5 by 2060, respectively, primarily south of the current extent. Expansion will be slight under both SSP1-2.6 and 179 km² under SSP5-8.5. By 2080, contraction is projected at 2,261 km² and 3,002 km² under SSP1-2.6 and SSP5-8.5, respectively, with minor expansions of 289 km² under SSP1-2.6 and 26 km² under SSP5-8.5.

The IPSL-CM6A-LR model predicts a contraction of the existing distribution area by 3,428 km², 3,533 km², 3,540 km², and 2,567 km² under SSP1-2.6 (2041–2060), SSP5-8.5 (2041–2060), SSP1-2.6 (2061–2080), and SSP5-8.5 (2061–2080), respectively, with minor expansions of 2 km² and 155 km² under SSP5-8.5 by 2060 and 2080, respectively.

Overall, for *L. monopetalum*, the contraction will surpass any expansion, predominantly affecting the parts south of the existing distribution. If expansion does occur, it will likely be a northward shift.

### Projected changes within protected areas

The MaxEnt predictions revealed that the potential habitats for *T. hirsuta* within protected areas in the investigated coastal region are expected to increase from 1,066 km² currently to 1,312–1,983 km² under the HadGEM3-GC31-LL model and 1,154–1,669 km² under the IPSL-CM6A-LR model. The ensemble model predictions are in accordance with that of MaxEnt, showing an increase in habitat suitability within protected areas from 12.23 km² currently to 2,375 km² and 2,379 km² under the HadGEM3-GC31-LL and IPSL-CM6A-LR models, respectively (Table 4).

For *O. vaginalis*, MaxEnt predictions under the HadGEM3-GC31-LL model exhibited a slight increase in suitable habitat within reserves, with suitability rising from 1,014 km² currently to 1,099 km² by 2080 under SSP5-8.5. The IPSL-CM6A-LR model predicts a decline in suitable habitat at 2060 (998 km² under lower and 703 km² under higher emission scenarios), but an increase by 1,295 km² and 1,031 km² under SSP1-2.6 and SSP5-8.5, respectively, by 2080. Ensemble models show a rise in suitable habitat under HadGEM3-GC31-LL, rising from 969 km² currently to 2,409 km² under SSP1-2.6 by 2060, and under IPSL-CM6A-LR, increasing by 2,164 km² under SSP5-8.5 by 2060 (Table 4).

For *L. monopetalum*, MaxEnt predicts a decline in suitable habitat within protected areas from 1,101 km² currently, with a decline of 350 km² and 466 km² by 2080 under SSP5-8.5 for HadGEM3-GC31-LL and IPSL-CM6A-LR, respectively. Ensemble model predictions corroborate this decrease in suitable extent (Table 4).

### Summary of projected habitat distribution and climate effects

Climate projections under various models and socio-economic pathways (SSP1-2.6 and SSP5-8.5) suggest varying impacts on the future distribution of suitable habitats for *T. hirsuta*, *O. vaginalis*, and *L. monopetalum*.

#### T. hirsuta

MaxEnt and ensemble models predict an overall **increase in suitable habitat**, particularly under SSP1-2.6, with expansions most prominent in the northwest. However, some contractions are expected in the central and eastern parts of its current range. By 2080, ensemble models project increases of up to 23,891 km². Within protected areas, suitable habitat is expected to rise significantly under both climate models.

#### O. vaginalis

Future projections indicate a **moderate increase in suitable habitat**, with both contraction and expansion occurring—mainly shifting northward. Ensemble models under both GCMs show growth in habitat extent, especially under SSP5-8.5, reaching up to 12,479 km² by 2080. Within protected areas, predictions vary slightly but generally show a positive trend by 2080, especially under ensemble models.

#### L. monopetalum

In contrast, *L. monopetalum* is projected to **experience significant habitat contraction** under all scenarios and models, with the greatest losses near its current coastal range. Although minimal northward expansion may occur, the overall trend indicates a loss of 83–87% of suitable area by 2080. This contraction is mirrored in protected areas, where habitat extent is expected to decline consistently.

**Table 4:** The area (in km^2^) of the protected area that regard suitable habitat for the studied species currently and under future climate scenarios based on MaxEnt and ensemble models predictions

Species	Model	Suitability class	Current	HadGEM3-GC31-LL				IPSL-CM6A-LR			
				2041–2060		2061–2080		2041–2060		2061–2080	
				SSP1-2.6	SSP5-8.5	SSP1-2.6	SSP5-8.5	SSP1-2.6	SSP5-8.5	SSP1-2.6	SSP5-8.5
*T. hirsuta*	MaxEnt	Unsuitable	7918	7672	7042	7001	7383	7830	7476	7678	7315
		Low	138	743	1101	1055	980	665	926	791	972
		Moderate	503	375	632	724	445	367	453	373	502
		High	425	194	209	204	176	122	129	142	195
	Ensemble	Unsuitable	7885	6711	6609	6675	6641	7090	6682	6672	6605
		Low	295	1047	1079	1158	1271	629	980	920	1322
		Moderate	130	792	856	615	710	899	1035	1077	647
		High	674	434	440	536	362	366	287	315	410
*O. vaginalis*	MaxEnt	Unsuitable	7970	7954	7969	7951	7885	7986	8281	7689	7953
		Low	465	375	634	377	482	486	587	475	420
		Moderate	424	520	105	209	196	400	49	97	127
		High	125	135	276	447	421	112	67	723	484
	Ensemble	Unsuitable	8015	6575	7414	7894	7196	7610	6821	7529	7770
		Low	453	1711	684	327	969	714	1348	787	401
		Moderate	411	403	417	336	281	389	556	391	407
		High	105	295	469	427	538	271	259	277	406
*L. monopetalum*	MaxEnt	Unsuitable	7883	8143	8203	8164	8634	8148	8304	8050	8518
		Low	526	408	492	515	289	464	535	506	367
		Moderate	374	321	236	244	61	286	115	315	99
		High	201	112	53	61	0	86	30	113	0
	Ensemble	Unsuitable	7872	8788	8729	8703	8745	8791	8816	8802	8725
		Low	652	150	211	231	202	152	139	142	221
		Moderate	382	46	44	50	37	41	29	40	38
		High	78	0	0	0	0	0	0	0	0

#### Overall trends

**Expansion** is most likely for *T. hirsuta* and *O. vaginalis*, particularly under SSP1-2.6 of the ensemble model outputs.

**Contraction** is a dominant trend for *L. monopetalum*, signaling a need for urgent conservation attention

**Protected areas** will remain important refuges, with models indicating varying degrees of future suitability across species.

## Discussion

### Model performance and evaluation

The outcomes of the ensemble model obtained in the current study were very accurate as indicated by all the measures recommended for assessing models’ performance. Nevertheless, the results demonstrated that MaxEnt performed and predicted well comparative to the ensemble approach in identifying important areas for the studied Egyptian wild species. These findings do not basically infer that MaxEnt is a more reliable modelling method comparative to the other techniques [17]. Nonetheless, it can be suggested that MaxEnt be recognized among the highly consistent and robust techniques for species distributions modelling, particularly in cases of limited occurrence records [43, 44, 49]. It could be simply applied to support in the identification and proposing of important conservation sites for key species.

It is worth noting that suggesting MaxEnt as a more reliable alternative to the ensemble approach is not intended to be inferred here. The ensemble model attained high accuracy in the present study, however MaxEnt produced consistent results across the investigated climate scenarios, where the outcomes of the investigated scenarios attained a similarity index that exceeded 0.90. Therefore, MaxEnt is deemed as a competent option comparative to the other complex, computationally intensive ‘black box’ approaches. Moreover, it is highly recommended for studies based on presence-only data, particularly for areas where the systematic survey through which obtaining species presence/absence data may not be always possible for logistical reasons. So, it is thought that in such circumstances MaxEnt can help in the promotion of the measures intended for rational and effective conservation actions.

Overall, the ensemble model showed higher AUC values compared to MaxEnt model in the present study. Both the single algorithm model and the ensemble model showed high accuracy and prediction performance. Generally, the ensemble and MaxEnt models exhibited alike trends in predicting the future distribution for the three studied species, *T. hirsuta*, *L. monopetalum*, and *O. vaginalis*. The ensemble model showed promising outcomes based on the the accuracy measures values comparative to the MaxEnt model. Both the MaxEnt and the ensemble models show high accuracy and performance, and similar trends in the predicting *O. vaginalis* distribution.

### Climatic changes potential effects on the mediterranean species

Ecological niche and SDMs are primal to climate change biology [50] through facilitating the studies of the potential effect of climatic changes on species distribution and geographic ranges [50–52]. Especially when data from reliable climatic models are integrated with information on land-use changes to assess the collective potential effects of these changes on species distribution [52]. With the development in the GCMs and the associated improvement in the scenarios of climatic changes, clearer depiction of the influence on species distribution can be obtained [53]. The assessment of models’ performance and the realization of their limitations are necessary to prevent misuse of models’ outcomes and avoid oversights in habitat prioritization for conservation and reserve design [54]. Plants vary in their reaction to climate changes depending mainly on their physiological or phenological characteristics [55]. The impacts of climatic changes are being remarked in the form of shift and change in species ranges [56].

The prediction of the explored species under current climatic conditions was investigated by Mahmoud et al. (2024) [57], and the summary of the outcomes related to the current potential distribution of these species are provided as supplementary material. The geographic extent of *T. hirsuta* is expected to respond differently under the diverse climatic change scenarios. Under the future condition the predicted distribution area of *T. hirsuta* in each of the studied timeframe changed among different future periods and SSPs comparative to the current distribution pattern. The MaxEnt predictions for the two climatic scenarios, HadGEM3-GC31-LL and IPSL-CM6A-LR, of the potential change in habitat suitability followed the same trend under the two SSPs scenarios (SSPs126 and SSPs585). The prediction revealed that the geographic spread of *T. hirsuta* will increase compared to current predictions by the time, the expansion of suitability area tends to the Libyan Desert into the northwest direction, and the expansion average will increase, and the contraction average will increase too. The trend of habitat range shifts due to climatic changes was also reported for other mountain plant species in North Africa and in the mountainous area in the Mediterranean [58–60]. Changes in climatic environmental variables such as bio8, bio9, bio1 and bio19 drive the change in the suitable area and distribution pattern of *T. hirsuta*.

According to the ensemble modelling outcomes of HadGEM3-GC31-LL and IPSL-CM6A-LR model, the potential changes in habitat suitability followed the same trend under the two emissions scenarios SSPs126 and SSPs585 during the two timeframes, 2041–2060 and 2061–2080, and all dispersal scenarios assessed. For *T. hirsuta* species, climatically suitability area will expand entirely under SSPs126 and SSPs585 emissions scenarios of all periods and full and limited dispersal scenarios. The spread of the suitability area tends to the northwest to Libyan Desert direction. The contraction of the suitability area will occur but with little rate. It seems that the unsuitable areas will be characterized by high temperature and low precipitation in the future. Both MaxEnt and the Ensemble models showed the same trends and same results. The prediction under the two general climate model scenarios forecast comparatively close outcomes as both show expansion of *T. hirsuta* suitable area distribution in the Libyan Desert direction.

The suitable habitat for *O. vaginalis* is estimated to increase in the near (2041–2060) and far future (2061–2080) predictions obtained by both MaxEnt and ensemble models under the scenarios of the HadGEM3-GC31-LL climate model. However, more expansion is projected by the ensemble model relative to that predicted by the MaxEnt model. Although under the scenarios of the IPSL-CM6A-LR GCM, the suitable areas are projected to decrease during the 2041–2060 period, a slight increase is projected on the long term (2061–2080 period) based on the MaxEnt predictions. Consistent projections were obtained by the ensemble model under the scenarios of the two general climate models revealing predicted expansion in the suitable distribution to northwest part of the Libyan desert.

Overall, under all climate scenarios a geographic shift in the distribution in the suitability area would slightly shift to the northwest direction, where the expansion rate is larger than the contraction rate of the suitability area under all future climatic scenarios.

Plants react to the fluctuation in the climate by changing their distribution through either migrating to higher latitudes or higher altitudes or combination of that in their quest for suitable environment conditions [61, 62]. The projected shifts in the suitable area of *L. monopetalum* under the SSP1-2.6 and SSP5-8.5 scenarios of the two general climate models over the short (2041–2060) and long term (2061–2080) and based on MaxEnt and ensemble models predictions revealed that the geographic distribution of *L. monopetalum* would shrink under the future conditions. The projected rise in temperature and decline in precipitation under these scenarios appears to be the most significant drivers leading to the future shrinkage of the species distribution range.

The loss of suitable habitats might have adverse consequences on species extent. Extreme drought and habitat loss tend to challenge the survival of *L. monopetalum* species, and this might be translated into negative rates of population expansion in the worst scenarios. Low growth rate and high death rate may lead to local extinctions [52, 63]. The warming in the Mediterranean area is anticipated to surpass the global rates by 25%, indicating that conservation priority should be given for the species inhabiting the arid sections of the Mediterranean area [64]. It was demonstrated that variations in precipitation levels resulting from future climate changes significantly influence the potential distribution and the habitat suitability of plant species worldwide [65]. Studies on the influence of the climatic changes in North Africa project that annual precipitation is probably to decrease by the end of the 21^st^ century [66, 67], which was supported by the REMO regional model that under the SRES A1B scenario predicted a potential decrease by more than 10% in rainfall in the area by 2050 [68]. Given that precipitation was revealed in the present study as the main factor influencing *L. monopetalum* distribution, proactive measures should be taken for the conservation of the remaining habitats of this species.

However, depending on their morphological and physiological features, plants may develop different responses and adaptation mechanisms to acclimatize to climatic changes [55]. According to Khanum et al. (2013), species with large ecological niche are assumed to be more adaptable to environmental changes than those exhibiting limited ecological range [69]. *L. monopetalum* can be recognized as a species with a narrow geographical niche and dispersal ability, which might reduce its capability to face climate change consequences, especially if the human-induced habitat fragmentation will provide barriers to its dispersal ability.

Egypt currently boasts a considerable and fairly representative protected area system, consisting of 50 protected areas [28]. However, the future effectiveness of these protected areas is highly dependent on how well species can adjust to the changing climate. Ensuring the continued representativeness of the studied species within the reserves over time will require an understanding of species’ reactions to climatic changes and proactive management to address emerging conservation challenges.

Based on the modelled distribution and key environmental drivers identified, targeted conservation strategies should focus on protecting habitats within high-suitability areas, particularly those influenced by coastal proximity and stable microclimates. Priority should be given to mitigating habitat loss from coastal development, preserving ecological corridors for dispersal, and monitoring populations in vulnerable fringe zones. Integrating these strategies into regional planning could significantly enhance the long-term viability of the species.

Conservation efforts should prioritize habitat restoration in degraded but high-potential areas, especially those near the coast where maritime influences create favorable conditions. Establishing protected areas or buffer zones in these regions could help minimize human disturbance. Additionally, engaging local communities in conservation activities and raising awareness about the species’ ecological role may foster long-term stewardship. Incorporating climate resilience into management plans will also be essential, given the species’ sensitivity to environmental changes.

## Conclusion

It could be concluded from the present study that the habitat preferences of the investigated species could be used for any future conservation or restoration program. The MaxEnt model and the ensemble models showed excellent performance and agreement in predicting potential future distribution of the investigated species. The projections developed in this study indicate that *Limoniastrum monopetalum* will experience a continuous decline in suitable habitat areas, increasing its danger of extinction under various climatic change scenarios. Therefore, immediate conservation and management strategies are recommended to safeguard this species. The distribution extent of *Thymelaea hirsuta* and *Ononis vaginalis* range is forecasted to shift northwestwardly. It is recommended to develop conservation and management plans for the investigated species along the Mediterranean coast to protect them from local risks and anthropogenic activities.

## Electronic supplementary material

Below is the link to the electronic supplementary material.

Supplementary Material 1

## Data Availability

The datasets generated during and/or analyzed during the current study are available from the corresponding author on reasonable request.
