# Supplementary material for: Predicting the future impact of climate change on the distribution of species in Egypt’s mediterranean ecosystems
Source: BMC Plant Biol. 2025 May 15;25:644. doi: 10.1186/s12870-025-06630-7 (PMC12079925; doi:10.1186/s12870-025-06630-7)
Supplement: Supplementary file 1 — Supplementary Material 1 [file 12870_2025_6630_MOESM1_ESM.docx]

**Supplementary Material**

**Table S1.** List of the environmental variables used in the study.

| Abbreviation | Variables | Units |
| --- | --- | --- |
| Climatic/Bioclimatic variables | | |
| bio1 | Annual mean temperature | °C |
| bio2 | Mean diurnal range (mean of monthly (max temp – min temp)) | °C |
| bio3 | Isothermality (P2/P7) ×100 | °C |
| bio4 | Temperature seasonality (standard deviation×100) | °C |
| bio5 | Max temperature of warmest Month | °C |
| bio6 | Min temperature of coldest Month | °C |
| bio7 | Temperature annual range (P5-P6) | °C |
| bio8 | Mean temperature of wettest quarter | °C |
| bio9 | Mean temperature of driest quarter | °C |
| bio10 | Mean temperature of warmest quarter | °C |
| bio11 | Mean temperature of coldest quarter | °C |
| bio12 | Annual precipitation | mm |
| bio13 | Precipitation of wettest Month | mm |
| bio14 | Precipitation of driest Month | mm |
| bio15 | Precipitation of seasonality (Coefficient of Variation) | mm |
| bio16 | Precipitation of wettest quarter | mm |
| bio17 | Precipitation of driest quarter | mm |
| bio18 | Precipitation of warmest quarter | mm |
| bio19 | Precipitation of coldest quarter | mm |
| Prec | Precipitation | mm |
| Tmax | Maximum temperature | °C |
| Tmin | Minimum temperature | °C |
| Tavg | Average temperature | °C |
| Topographic variables | | |
| Alt | Elevation | m |
| Soil variables | | |
| Bulk density | Bulk density | cg/cm3 |
| Clay | Clay content | g/kg |
| Coarse fragments | Coarse fragments | cm3/dm3 |
| Nitrogen | Nitrogen | cg/kg |
| Sand | Sand | g/kg |

**Table S1.** **(Continued)**

| Abbreviation | Variables | Units |
| --- | --- | --- |
| Soil variables | | |
| Silt | Silt | g/kg |
| Carbon density | Organic carbon density | g/dm^3^ |
| Cation exchange | Cation exchange capacity (at pH 7) | mmol(c)/kg |
| pH | Soil pH | pH × 10 |
| Habitat | | |
| Habitat | Habitat types | - |
| Proximity to sea | Distance to coastline | km |

**Table S2.** Correlation matrix of the Spearman correlation analysis between combinations of the environmental variables used in modelling the distribution of the studied species. For abbreviations, See Table S1.

** Correlation is significant at the 0.01 level (2-tailed).

* Correlation is significant at the 0.05 level (2-tailed).

| *Thymelaea hirsuta* | | | | | | | | | | |
| --- | --- | --- | --- | --- | --- | --- | --- | --- | --- | --- |
|  | **bio1** | **bio19** | **bio8** | **bio9** | **Alt** | **tmax** | **Distance** | **Habitat** |  |  |
| bio1 | 1.000 |  |  |  |  |  |  |  |  |  |
| bio19 | .489^**^ | 1.000 |  |  |  |  |  |  |  |  |
| bio8 | .595^**^ | .227^**^ | 1.000 |  |  |  |  |  |  |  |
| bio9 | .656^**^ | .213^**^ | .196^**^ | 1.000 |  |  |  |  |  |  |
| Alt | -.591^**^ | -.543^**^ | -.514^**^ | -.132^*^ | 1.000 |  |  |  |  |  |
| t_max_ | .196^**^ | -.495^**^ | .230^**^ | .408^**^ | .326^**^ | 1.000 |  |  |  |  |
| Distance | -.362^**^ | -.751^**^ | -.282^**^ | .111^*^ | .427^**^ | .674^**^ | 1.000 |  |  |  |
| Habitat | -.307^**^ | -.054 | -.493^**^ | -.066 | .227^**^ | -.035 | .097 | 1.000 |  |  |
| *Limoniastrum monopetalum* | | | | | | | | | | |
|  | **bio18** | **bio19** | **bio9** | **Clay** | **Caorse** | **nitrogen** | **silt** | **Alt** | **Distance** | **Habitat** |
| bio18 | 1.000 |  |  |  |  |  |  |  |  |  |
| bio19 | .620^**^ | 1.000 |  |  |  |  |  |  |  |  |
| bio9 | .150 | .587^**^ | 1.000 |  |  |  |  |  |  |  |
| Clay | .071 | .175 | .427^**^ | 1.000 |  |  |  |  |  |  |
| Caorse fragment | .332^**^ | .413^**^ | .401^**^ | .721^**^ | 1.000 |  |  |  |  |  |
| nitrogen | .004 | .475^**^ | .381^**^ | .478^**^ | .561^**^ | 1.000 |  |  |  |  |
| Silt | .196 | .410^**^ | .422^**^ | .511^**^ | .693^**^ | .712^**^ | 1.000 |  |  |  |
| Alt | .276^*^ | .206 | .433^**^ | .492^**^ | .515^**^ | .071 | .356^**^ | 1.000 |  |  |
| Distance | -.164 | -.014 | .489^**^ | .450^**^ | .422^**^ | .079 | .311^**^ | .641^**^ | 1.000 |  |
| Habitat | .263^*^ | -.055 | -.166 | -.167 | -.164 | -.424^**^ | -.265^*^ | -.077 | -.152 | 1.000 |
| *Ononis vaginalis* | | | | | | | | | | |
|  | **pH** | **bio19** | **bio3** | **bio8** | **bio9** | **Alt** | **Prec** | **Distance** | **Habitat** |  |
| pH | 1.000 |  |  |  |  |  |  |  |  |  |
| bio19 | .463^**^ | 1.000 |  |  |  |  |  |  |  |  |
| bio3 | -.112 | .014 | 1.000 |  |  |  |  |  |  |  |
| bio8 | .059 | -.139 | .523^**^ | 1.000 |  |  |  |  |  |  |
| bio9 | .156 | .589^**^ | .317^*^ | .252^*^ | 1.000 |  |  |  |  |  |
| Alt | .057 | .168 | .470^**^ | -.008 | .355^**^ | 1.000 |  |  |  |  |
| Prec | .025 | .407^**^ | -.716^**^ | -.731^**^ | -.071 | -.378^**^ | 1.000 |  |  |  |
| Distance | .035 | -.005 | .611^**^ | .240 | .437^**^ | .468^**^ | -.516^**^ | 1.000 |  |  |
| Habitat | -.074 | .210 | -.339^**^ | -.614^**^ | -.039 | -.269^*^ | .407^**^ | -.328^**^ | 1.000 |  |

**Table S3.** The importance of predictor bioclimatic variables to the potential distribution of *Thymelaea hirsuta, Ononis vaginalis* and *Limoniastrum monopetalum* species. Correlated variables with variance inflation factor (VIF) values > 5, and correlation threshold of 0.75 were removed to avoid problems related to collinearity. **Variables importance**

| Species | Variables | VIF | Variables importance (%) | |
| --- | --- | --- | --- | --- |
|  |  |  | **Maxent** | **Ensemble** |
| *T. hirsuta* | Distance to coastline | 3.90 | 50.11 | 39.4 |
|  | bio9 | 1.67 | 18.52 | 29 |
|  | bio8 | 2.68 | 17.26 | 15.1 |
|  | bio1 | 2.56 | 6.88 | 27.7 |
|  | Habitat type | 1.90 | 4.72 | 0.6 |
|  | bio19 | 1.36 | 1.74 | 2 |
|  | tmax | 2.57 | 0.59 | 22 |
|  | Alt | 3.64 | 0.21 | 2.9 |
| *O. vaginalis* | Distance to coastline | 2.45 | 84.04 | 83.8 |
|  | Habitat type | 3.60 | 9.11 | 5.7 |
|  | bio9 | 2.75 | 3.08 | 20 |
|  | bio19 | 1.44 | 1.33 | 3.3 |
|  | pH | 3.10 | 1.13 | 3.1 |
|  | bio8 | 3.50 | 0.53 | 10 |
|  | bio3 | 1.87 | 0.5 | 7.2 |
|  | Alt | 2.23 | 0.16 | 6.8 |
|  | Prec | 4.50 | 0.09 | 3 |
| *L. monopetalum* | Distance to coastline | 2.35 | 82.54 | 83.8 |
|  | Habitat type | 1.40 | 10.40 | 3.2 |
|  | bio19 | 3.68 | 2.54 | 27.3 |
|  | bio9 | 3.68 | 1.98 | 9.4 |
|  | bio18 | 2.32 | 1.10 | 1.8 |
|  | Clay | 4.42 | 0.28 | 6 |
|  | Alt | 2.18 | 0.07 | 7.9 |
|  | Silt | 3.24 | 0.89 | 1.9 |
|  | Coarse Fragment | 3.58 | 0.13 | 5.3 |
|  | Nitrogen | 2.26 | 0.03 | 2.7 |

Logistic probability of presence/ suitability

*Thymelaea hirsuta*

*Ononis vaginalis*

*Limoniastrum monopetalum*


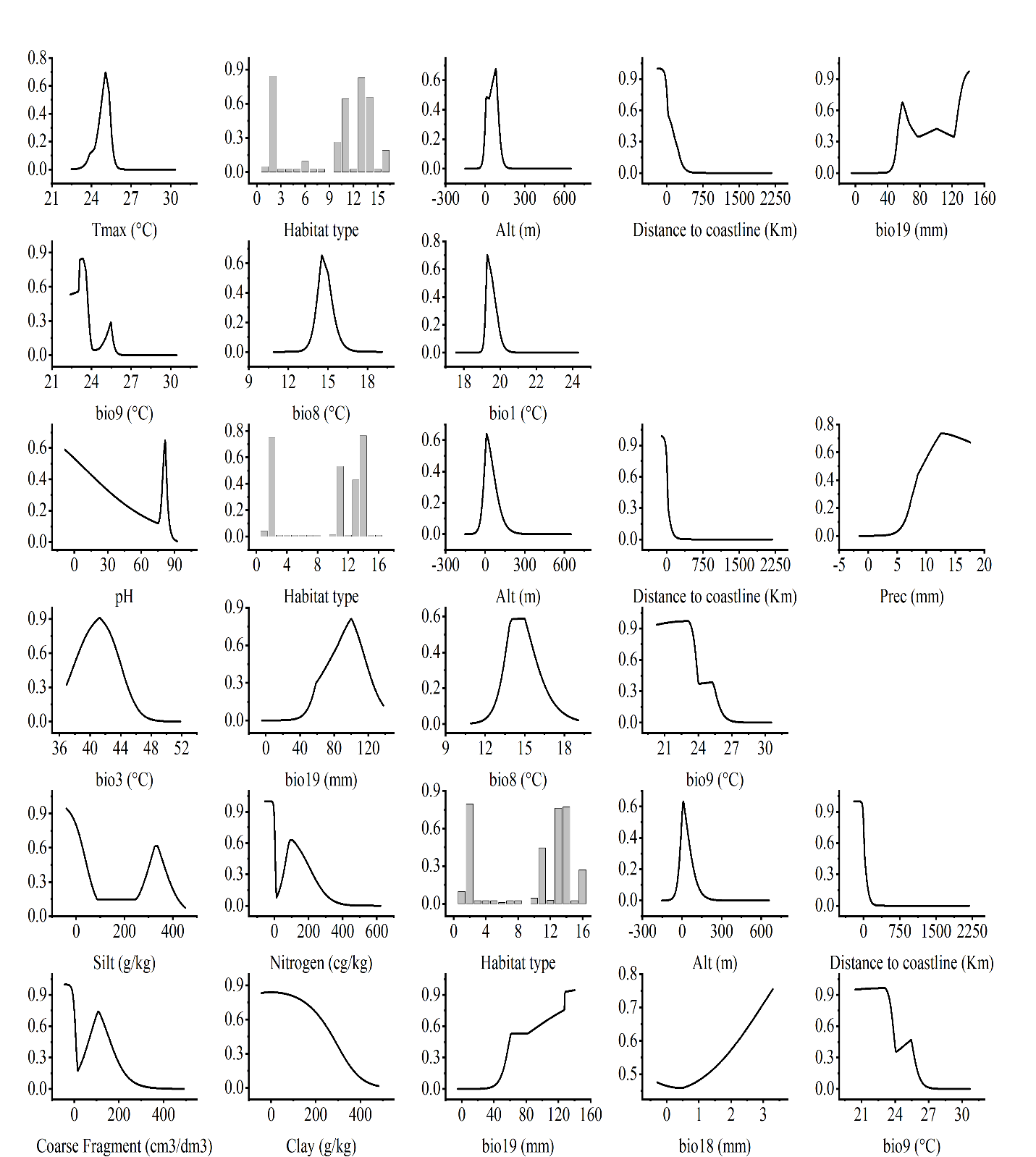


**Fig. S1.** Response curves of important environmental variables used in the Maxent distribution model of the studied species. For abbreviations, See Table S1.

**Fig. S2.** Response curves of important environmental variables used in the ensemble distribution model of the studied species. For abbreviations, See Table S1.


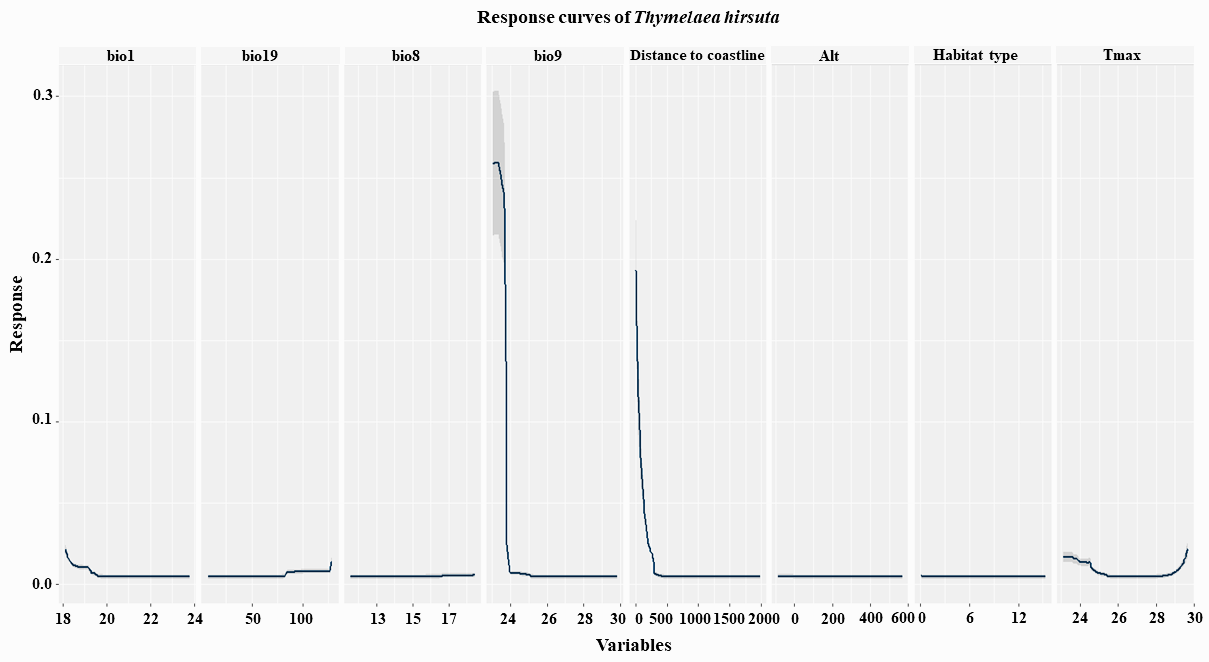

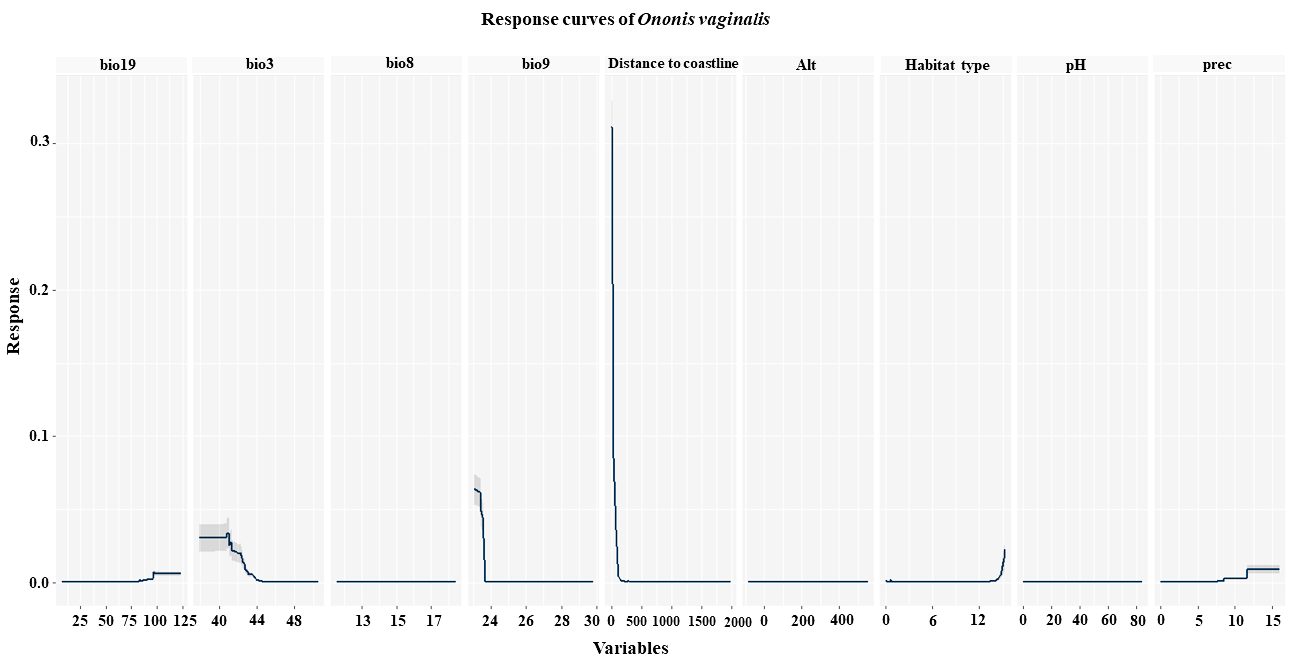

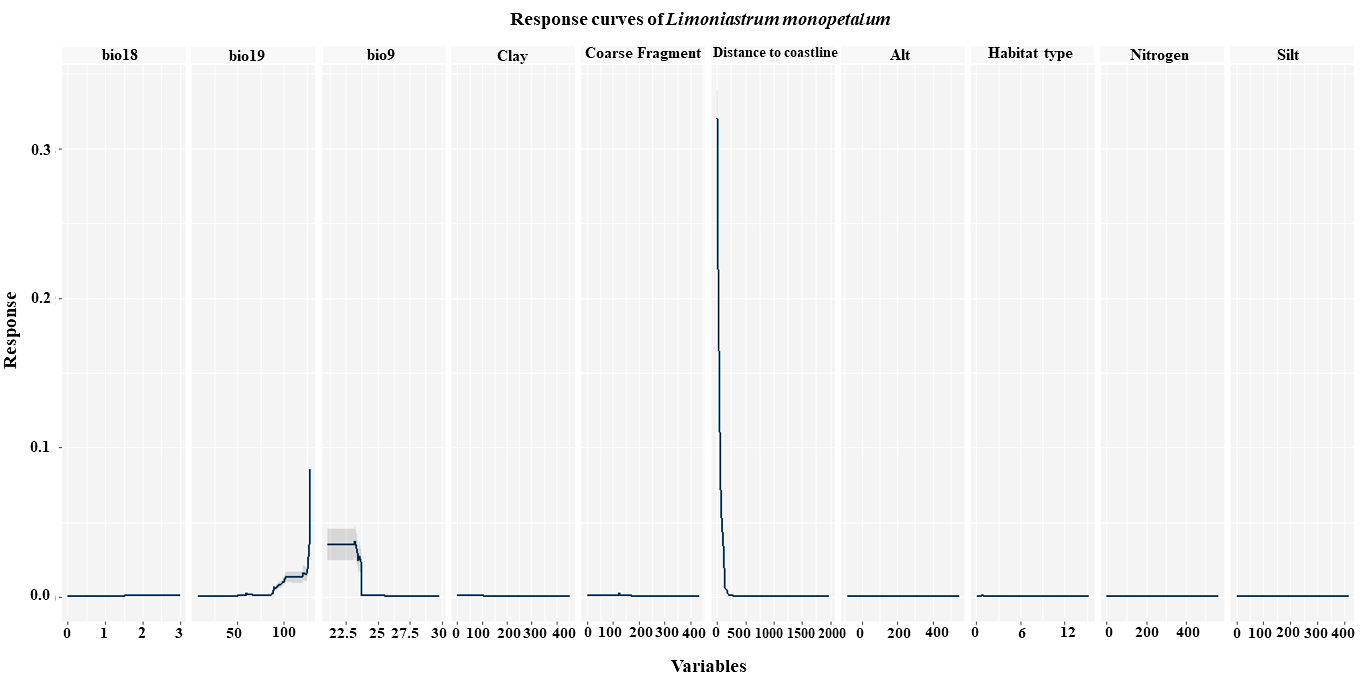


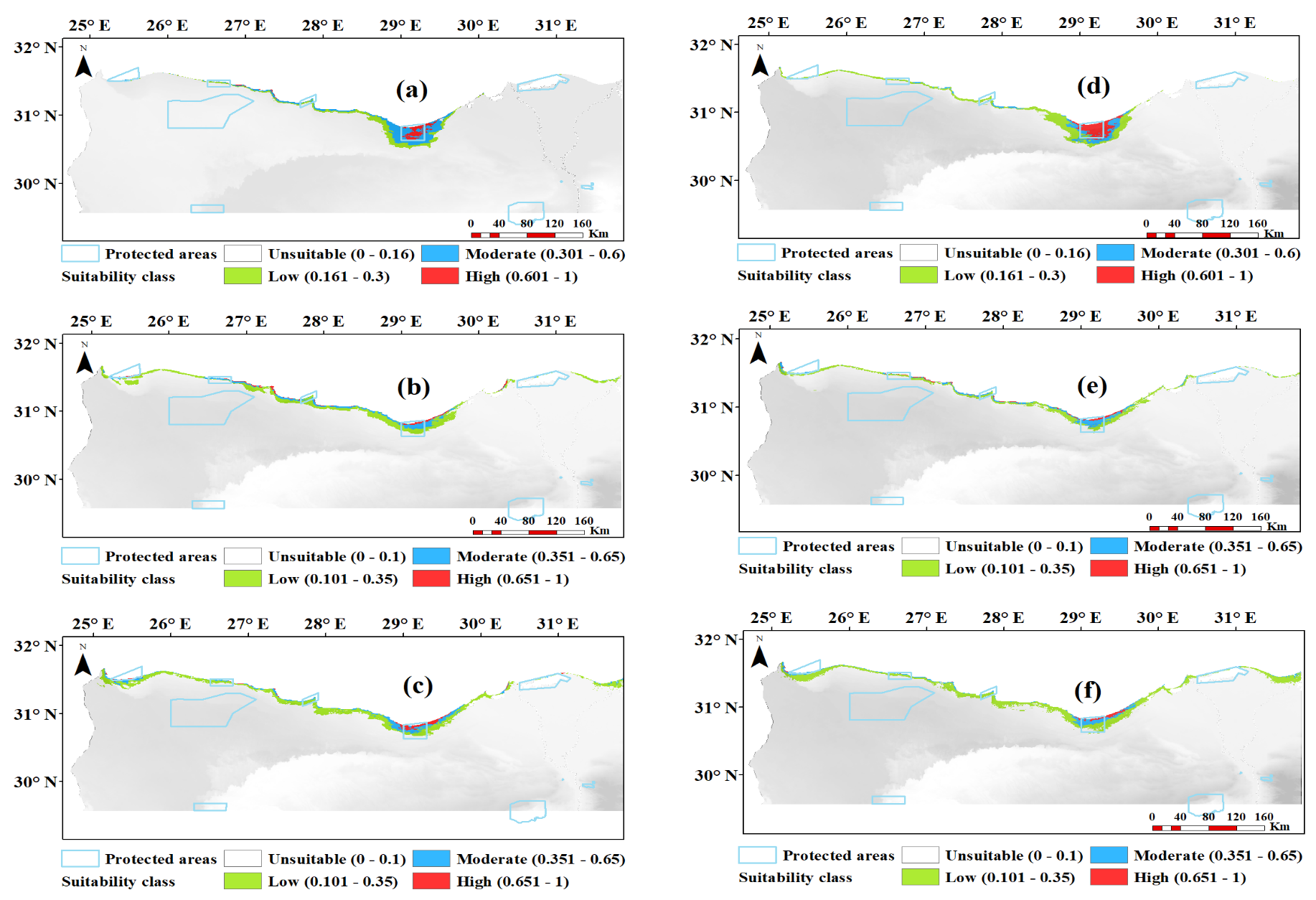
**Fig. S3.** The Predicted potential distribution of the three species under current climate conditions based on Maxent model and the Ensemble models: (a) and (d) for *Thymelaea hirsuta*, (b) and (e) *Ononis vaginalis* and (c) and (f) *Limoniastrum monopetalum*.
